# Fluorescent Nano-Probes to Image Plant Cell Walls by Super-Resolution STED Microscopy

**DOI:** 10.3390/plants7010011

**Published:** 2018-02-06

**Authors:** Gabriel Paës, Anouck Habrant, Christine Terryn

**Affiliations:** 1 FARE Laboratory, INRA, Université de Reims Champagne-Ardenne, 2 Esplanade Roland Garros, 51100 Reims, France; anouck.habrant@inra.fr; 2 Plateforme d’Imagerie Cellulaire et Tissulaire (PICT), Université de Reims Champagne-Ardenne, 51 rue Cognacq-Jay, 51100 Reims, France; christine.terryn@univ-reims.fr

**Keywords:** super-resolution microscopy, fluorescent probe, STED, plant cell wall, lignocellulose, poplar

## Abstract

Lignocellulosic biomass is a complex network of polymers making up the cell walls of plants. It represents a feedstock of sustainable resources to be converted into fuels, chemicals, and materials. Because of its complex architecture, lignocellulose is a recalcitrant material that requires some pretreatments and several types of catalysts to be transformed efficiently. Gaining more knowledge in the architecture of plant cell walls is therefore important to understand and optimize transformation processes. For the first time, super-resolution imaging of poplar wood samples has been performed using the Stimulated Emission Depletion (STED) technique. In comparison to standard confocal images, STED reveals new details in cell wall structure, allowing the identification of secondary walls and middle lamella with fine details, while keeping open the possibility to perform topochemistry by the use of relevant fluorescent nano-probes. In particular, the deconvolution of STED images increases the signal-to-noise ratio so that images become very well defined. The obtained results show that the STED super-resolution technique can be easily implemented by using cheap commercial fluorescent rhodamine-PEG nano-probes which outline the architecture of plant cell walls due to their interaction with lignin. Moreover, the sample preparation only requires easily-prepared plant sections of a few tens of micrometers, in addition to an easily-implemented post-treatment of images. Overall, the STED super-resolution technique in combination with a variety of nano-probes can provide a new vision of plant cell wall imaging by filling in the gap between classical photon microscopy and electron microscopy.

## 1. Introduction

The implementation of a super-resolution microscopy technique for imaging plant cell walls is a new topic with very few studies [1], whereas the need for accessing nanometer (nm)-resolution is well known. In fact, the investigation of the nanoscale lignocellulosic architecture has already been conducted in order to correlate structural modifications with enzymatic digestibility [2] by comparing the mechanisms of different enzymes [3] through the combination of, for example, different microscopy and tomography analyses [4]. However, all of these studies used electron microscopy or atomic force microscopy techniques, which are complex techniques regarding sample preparation and/or image analysis. Only one recent application of super-resolution technology to study cellulose orientation in onion cells has been published [5].

Several methods exist to reach nm resolution using photon microscopy. They are all based on the possibility of switching fluorescent dye molecules on and off into a dark state, whether by using a targeted switching approach (depletion wavelength), or a stochastic approach (photoactivable specific fluorophores) [6]. In the first case, by selectively depleting the signal of fluorescence emission at the outer regions of a focal spot, the microscope detects only the fluorescence light being emitted from the molecules at the center of the focal spots. Thus, it becomes possible to generate much sharper images than in confocal microscopy, theoretically down to 20 nm for lateral resolution [7].

**Figure 1:**
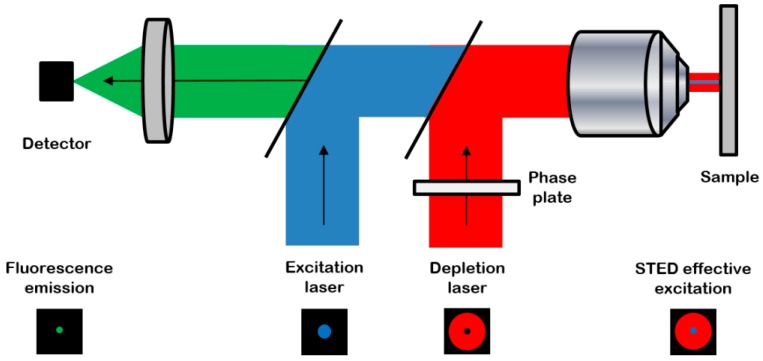
The principle of a stimulated emission depletion (STED) microscope (from reference [9]): the system combines the excitation laser (blue) with a depletion laser (red) passing through a phase plate to be modeled in “doughnut-shape” in the focal plane. The resulting excitation is an overlap of the two beams, leading to a high-resolution probe scanning the sample. Then the resulting effective detected fluorescence emission (green) is collected with high spatial and axial resolutions. Reproduced with permission from Journal of Internal Medicine published by Springer.

Stimulated emission depletion (STED) microscopy is a super-resolution microscopy method based on fluorescence confocal imaging, in which images are acquired by scanning a focused light spot over a region of interest and collecting the fluorescence sequentially, pixel by pixel [8] (Figure 1).

Advantages are: high resolution (ca. 50 nm in lateral *xy* and ca. 130 nm in axial *z* corresponding to a ca. 3-fold improvement in comparison to confocal microscopy [10]) without any complex additional post-processing; fast image acquisition (up to several images per second); possibility of choosing fluorophores over a wide type range (protein or small organic dyes) and spectral range enabled by several different STED lasers in one instrument. Limitations regard essentially the fluorophores: the depletion laser works fine with bright and stable fluorescent reporters that are neither turned on by the STED laser nor photobleached by it, and their emission spectrum must fit the depletion laser wavelength. A high-intensity STED laser can also photo-damage living cells, but non-living lignocellulose samples of low water content are much more resistant than living cells and less prone to photo-degradation.

STED microscopy is now routinely used in biomedical studies to study living cells [6,11]. In order to assay the interest of STED microscopy for imaging thin plant samples, we prepared poplar wood sections, whose ultrastructure has already been extensively imaged by photon and electron microscopy. Here, in order to specifically image the localization of lignin, poplar sections were incubated with a polyethylene-glycol (PEG)-based fluorescent probe, known to interact with lignin motifs [12]. Both confocal and STED microscopy were carried out simultaneously on the same sample, followed by a comprehensive image analysis to evaluate the difference between confocal and STED microscopy.

## 2. Results

### 2.1. Sample Preparation

The hydrolysis efficiency of plant biomass is mainly related to the enzyme accessibility [13]. In order to explore plant cell wall porosity, fluorescent probes [14] can advantageously be used [15]. Thus, 60-µm-thickness poplar sections were prepared and incubated with a fluorescent probe, a PEG-rhodamine (PEG-R). An interesting demonstrated property of the PEG molecule is its ability to bind to lignin motifs [12,16,17], thus simultaneously highlighting the cell wall architecture and lignin accessibility. PEG-R maximum excitation and emission wavelengths were measured at 544 nm and 576 nm, respectively (Figure S1). PEG-R average molecular weight (MW) and hydrodynamic radius (*R*_H_) were also determined, as previously reported [18]; they were 19.9 kDa (dispersity of 1.1) and of 3.2 nm, respectively. The measured MW is in agreement with that indicated by the supplier, which is 20 kDa. The size of this fluorescent probe is relevant to investigate the accessibility of plant cell walls since it is close to the *R*_H_ of commonly used cellulases, which degrade cellulose in the plant cell wall [18,19].

**Figure 2:**
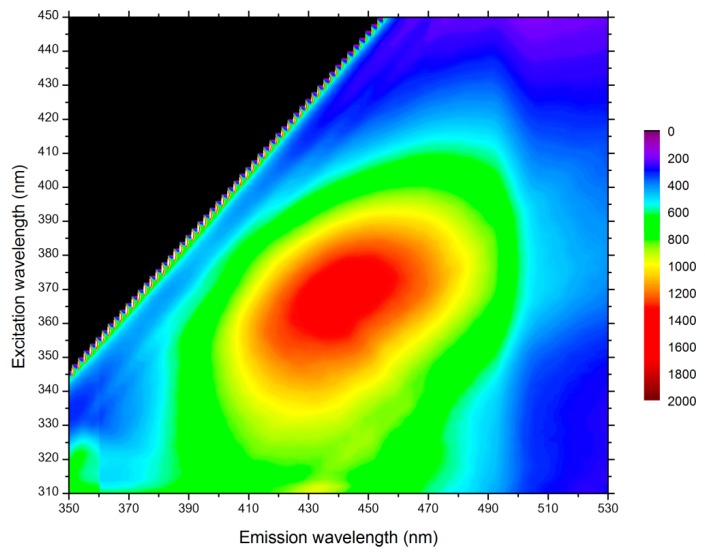
Autofluorescence contour map of poplar sample thin section. Maximum excitation occurs for wavelengths between 360 and 370 nm.

STED microscopy was possible only if no autofluorescence of poplar samples occurred at the depletion laser wavelength. Three different wavelengths were available on the STED microscope: 592 nm, 660 nm, and 775 nm. The infra-red depletion laser at 775 nm was selected, since the fluorescence signal from lignin and other aromatic compounds in the plant cell wall exhibited a maximum at an excitation of 360–370 nm and was thus negligible at 775 nm (Figure 2).

### 2.2. Image Acquisition and Analysis

**Figure 3:**
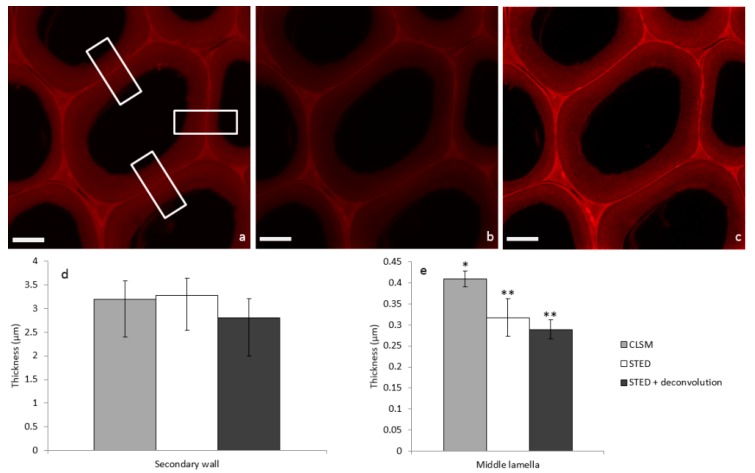
Images of poplar sections incubated with PEG-R and acquired in the same area by (**a**) CLSM, (**b**) STED, and (**c**) STED + deconvolution (scale bar: 5 µm). Corresponding mean thickness of (**d**) secondary cell wall and (**e**) middle lamella are extracted from Gaussian mean intensity (Supplementary File) profile in rectangle areas (means from *n* = 3, Mann-Whitney test indicates two separate groups marked by * and ** with a statistical difference *p* < 0.05).

In order to illustrate STED image properties, a systematic comparison of confocal laser scanning microscopy (CLSM), STED, and STED + deconvolution modes was performed on the same poplar sample incubated with PEG-R. First, considering the confocal image (Figure 3a), fluorescence due to PEG-R was mainly localized in the middle lamella (ML), which cements adjacent cells, while the secondary wall (SW) was less fluorescent. This is in accordance with the heterogeneous lignin distribution observed in poplar cells by UV micro-spectrophotometry [20]. In fact, during plant growth, lignin is deposited within the polysaccharide cell wall framework by filling inter-lamellar voids [21]. Comparison of the SW in the three modes (Figure 3a–c) does not seem to highlight dramatic structural differences. Overall thickness is not statistically different, being around 3 µm (Figure 3d). On the contrary, the ML thickness in the STED or STED + deconvolution modes appears to be 0.3 µm, in comparison to 0.4 µm in the confocal mode (Figure 3e). This former value is in accordance with previous measurements obtained by electron microscopy [22], thus indicating that the size of fine structures like the ML can be overestimated using confocal imaging alone, while the STED mode increases the resolution, thus giving more accurate measurements.

**Figure 4:**
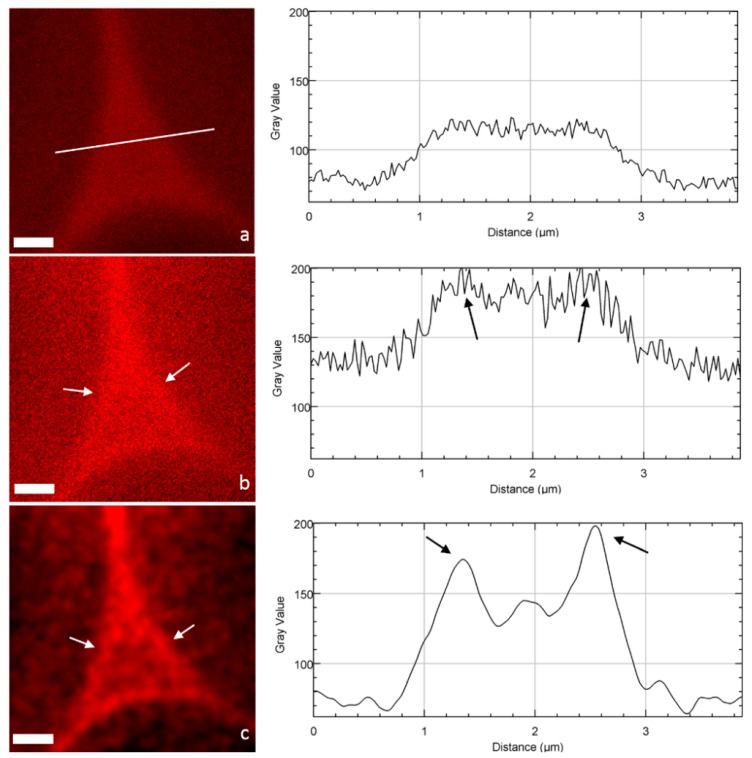
Images of cell corners and the corresponding intensity profile (line) acquired in the same area by (**a**) CLSM, (**b**) STED, and (**c**) STED + deconvolution (scale bar: 1 µm). Fine structures corresponding to the ML are not visible in the CLSM image, but become detected in the STED mode (arrows) and more precisely in the STED with deconvolution mode (arrows), with a large increase in amplitude and an improvement in the signal-to-noise ratio.

When focusing on cell corners (CCs), important differences can be drawn between CLSM and STED images (Figure 4). STED images appear less blurred, which allows a much better identification and definition of the ML and CCs. The impact of deconvolution on STED images was also very high: noise disappears by assigning all recorded intensities to the location from which they originate (the principle of deconvolution process), so that highly contrasted structures were obtained. To quantitatively demonstrate this visual observation, fluorescence intensity profiles [23] of the pixels along a line crossing the CC were drawn (Figure 4a–c). Both confocal and STED profiles seem noisy with no clear trend. In contrast, the STED-deconvolved image shows a well-defined profile corresponding to the CC architecture: two maximum intensity peaks corresponding to the ML of two adjacent cells and a minor peak in between, probably revealing the heterogeneity of lignin distribution in the CC structure, in accordance with the architecture imaged by transmission electron microscopy [24,25]. Interestingly, the use of fluorescent probes like PEG-R, which interacts with lignin, gives a chemical mapping of lignin distribution in the cell wall, revealing a high lignin density in CCs and to a lesser extent in the primary and secondary cell walls, in accordance, for example, with chemical imaging realized by confocal Raman microscopy [26]. Overall, although it does not reach the high resolution of electron microscopy [24], STED microscopy and in particular the deconvolution process provide images with largely reduced signal-to-noise ratios, and allows the easier identification of plant cell wall fine structures.

## 3. Discussion

In this study, the advantages of STED microscopy over conventional confocal microscopy to image plant cell walls has been clearly demonstrated. Interestingly, the STED technique provides a gain in image resolution when used in combination with deconvolution treatment. Even if the STED contrast and resolution cannot reach those of transmission electron microscopy [24], STED benefits from the simplicity of sample preparation since even relatively thick specimens (several tens of µm) can be observed, contrary to transmission electron microscopy, which requires ultra-thin samples (10–100 nm thickness) [24]. Moreover, STED microscopy is versatile since different types of chemical features can be observed depending on the type of extrinsic fluorescent probes that are used for imaging.

As a proof of concept, we used PEG-R, which has a demonstrated affinity for lignin, to reveal the localization of lignin in the cell wall in addition to the heterogeneous layering of lignin in the cell wall. The localization of PEG-R was similar to that already characterized for lignin, demonstrating that the fluorescent probe accessibility was not restrained. Rhodamine seems to be a relevant and cheap fluorophore that can be exploited in STED microscopy for plant imaging. The use, for example, of carbohydrate binding modules (CBMs) which can have variable affinity towards cellulose (amorphous or crystalline) and hemicellulose (main-chain type and decoration) should provide the ability to more precisely pinpoint features related to these polysaccharides. Another advantage is that PEG and CBM probes are available commercially at relative cheap prices; PEG can even be purchased with a rhodamine fluorophore attached, while CBMs, as proteins, can be easily conjugated [27,28]. In addition to other specific probes such as inactivated enzymes [29], we predict that, similar to antibodies which are already available with a large repertoire of affinities, protein-based fluorescent probes [14] will be relevant to the investigation of plant development, plant decay by fungi, and the effect of wood treatment on physical and chemical properties. In particular, there is a need to acquire plant cell wall data in high resolution and in three spatial dimensions in order to better understand the features controlling biosynthesis and deconstruction processes [4,30]. In light of this, we predict that STED microscopy will be an important tool among super-resolution techniques used today, validated by the recent introduction of a STED-based new super-resolution technique [31].

## 4. Materials and Methods

### 4.1. Fluorescent Probe Characterization

PEG-rhodamine (PEG-R) of 20,000 Da (reference PSB-2262 from Creative PEGWorks, Durham, NC, USA) was selected as the fluorescent probe. The absolute MW and *R*_H_ of PEG-R was determined by size exclusion chromatography and light scattering (SEC-MALS) analysis. To summarize, 150 µL of 2 mg/mL probe diluted in 50 mM sodium nitrate buffer were injected at 0.6 mL/min on a Shodex KW 802.5 column equilibrated at 30 °C and connected to an HPLC system (Waters 717), equipped as follows: degas, UV-visible detector (Waters 2996), multi-angle static light scattering (MALS) detector DAWN HELEOS II (Wyatt, Santa-Barbara, CA, USA), dynamic light scattering detector DynaPro NanoStar Waters 2414 (Waters, Milford, MA, USA), refraction index detector (Waters 2414). Analysis of the chromatogram was performed with ASTRA 6.1 software (Wyatt).

### 4.2. Poplar Sample Preparation for Microscopy

**Figure 5:**
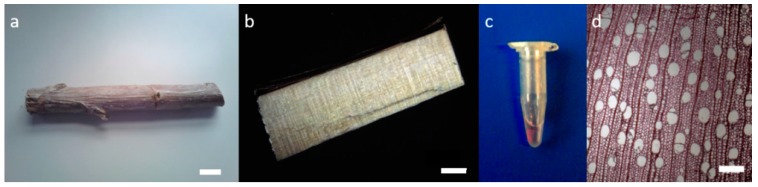
Preparation steps for the poplar sample thin sections: (**a**) Raw poplar wood (scale bar: 2 cm); (**b**) poplar fragment (scale bar: 2 mm); (**c**) poplar section with a thickness of 60 µm incubated with the PEG-R fluorescent probe in a plastic tube; (**d**) poplar section observed in photon microscopy (scale bar: 0.2 mm).

Poplar wood was obtained from Dorsk B1F + 41 clone 200. Transverse sections with a thickness of 60 µm were cut using a microtome equipped with disposables blades (Microm Microtech HM360, Brignais, France). Sections were incubated for 72 h at room temperature in 0.01% w/v PEG-R in 30 mM phosphate buffer, pH 6.0. Sample preparation steps are presented in Figure 5. Sections were mounted between a cover glass and a #1.5H coverslip using glycerol as the mounting medium, since its refractive index is very close to that of the immersion oil used for the dedicated STED objective (see below). A fluorescence contour map of the poplar sample was performed with a Jasco FP-8300 Instrument (Jasco, Lisses, France). Acquisition parameters were as follows: the range/precision for excitation and emission were 250–800 nm/2 nm and 260–850 nm/1 nm, respectively; the excitation and emission bandwidth was 2.5 nm; the scan speed was 1000 nm/min; the sensitivity (gain) was 300 V. Spectral acquisition and analysis were performed using Jasco Spectra Manager software (Jasco, Lisses, France).

### 4.3. Confocal and STED Imaging

A Leica TCS SP8 microscope was equipped with an STED laser and an STED 100x/NA 1.4 oil objective, with a working distance of 90 μm. The selected excitation laser was 552 nm for the imaging sample containing PEG-R; the depletion laser was 775 nm, set at a power of 1% of 1.9 W. Hybrid detectors were used with a detection window of 560–660 nm. Image acquisition parameters were: scan speed 400 Hz, image size 1024 × 1024 pixels, 8-bit color depth. A deconvolution step was performed to improve the image resolution. Briefly, by using sub-resolution beads, it was possible to determine the response system called Point Spread Function (PSF). With a known PSF, it became possible to computationally perform the deconvolution of the acquired signal to improve the signal-to-noise ratio, and thus the image resolution. Image deconvolution was performed using Huygens software implemented in LAS X Leica software. Image analysis and quantification were carried out in Fiji [32]. For each image, a mean of 100 intensity profiles (regions of interest are depicted as rectangles in Figure 3) were extracted. Profiles were fitted by a Gaussian curve using Matlab software, and sizes of ML and SW were given by the full width at half maximum (FWHM).

## Supplementary Materials

The following are available online at http://www.mdpi.com/2223-7747/7/1/11/s1, Figure S1: Autofluorescence contour map of PEG-R probe.
