# Supplementary material for: Fluorescent Nano-Probes to Image Plant Cell Walls by Super-Resolution STED Microscopy
Source: Plants (Basel). 2018 Feb 6;7(1):11. doi: 10.3390/plants7010011 (PMC5874600; doi:10.3390/plants7010011)
Supplement: Supplementary file 1 [file plants-07-00011-s001.pdf]

Fluorescent Nano-Probes to Image Plant Cell Walls by Super-Resolution STED Microscopy

Gabriel Paës *, Anouck Habrant and Christine Terryn

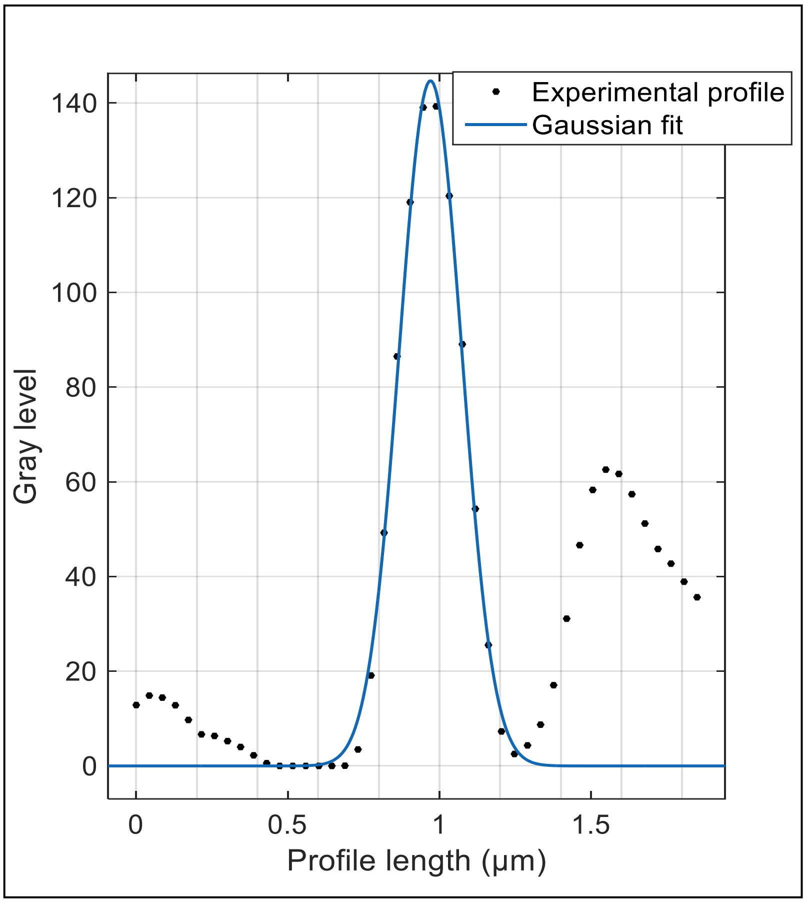

Figure S1. Gaussian fit of mean intensity profile measured in rectangle areas (see Figure 3).
